# Psilosis or "Sprue."

**Published:** 1899-06

**Authors:** 


**Psilosis or "Sprue."** By George Thin, M.D. Second Edition. Pp. xii., 270. London: J. & A. Churchill. 1897.

Dr. Thin in this book gives a full account of this disease, for the differentiation of which from other forms of tropical diarrhœa and chronic dysentery we have mainly to thank his clinical acumen. His observations seem to establish the existence of psilosis as a distinct disease. The further question whether some varieties of so-called hill-diarrhœa and of diarrhœa alba met with in India are identical with psilosis is still uncertain, but now that the symptoms of psilosis have been differentiated this point should soon be decided. The exciting cause of the disease has still to be discovered. There is an interesting review of the literature, a chapter on the pathology of the disease, and a full description of the treatment—first advocated by the author,—which has been attended by successful results. A number of cases are reported in an Appendix. The book is written in a clear and interesting style, and gives the reader full information on the subject, which is of practical importance, as any medical man may be called upon to treat such a case in a patient who has lived in tropical countries. Three coloured plates of the appearance of the tongue will be found useful, as an early and characteristic sign of psilosis is the alteration of this organ.

